# Supplementary material for: Human-Borne Pathogens: Are They Threatening Wild Great Ape Populations?
Source: Vet Sci. 2022 Jul 13;9(7):356. doi: 10.3390/vetsci9070356 (PMC9323791; doi:10.3390/vetsci9070356)
Supplement: Supplementary file 1 [file vetsci-09-00356-s001.zip › vetsci-1799582-supplementary.pdf]

Table S1. Estimated size of current great ape populations globally.

Country	Status	*Gorilla beringei*	*Gorilla gorilla*	*Pan troglodytes*	*Pan paniscus*	*Pongo abelii*	*Pongo pygmaeus*	Reference(s)
**Cameroon**	Total	–	34,700	23,800	–	–	–	[122,123]
	Habituated	–	100	Unknown	–	–	–	
**Central African Republic**	Total	–	Unknown	3,700	–	–	–	[124]
	Habituated	–	Unknown	Unknown	–	–	–	
**Côte d'Ivoire**	Total	–	–	1,095	–	–	–	[125]
	Habituated	–	–	150	–	–	–	[126]
**Democratic Republic of the Congo**	Total	4,083	189,600	200,000	20,000	–	–	[122,123,127,128]
	Habituated	339		400	Unknown	–	–	
**Gabon**	Total	–	85,300	Unknown	–	–	–	[122,123]
	Habituated	–	100	100	–	–	–	[129]
**Gambia**	Total	–	–	–	–	–	–	[125]
	Habituated	–	–	–	–	–	–	
**Guinea Bissau**	Total	–	–	1,908	–	–	–	[125]
	Habituated	–	–	Unknown	–	–	–	
**Indonesia**	Total	–	–	–	–	6,660/14,613[2]	54,000	[1,32,130,131]
	Habituated	–	–	–	–	Unknown	Unknown	
**Republic of Congo**	Total	–	6300	56,000	–	–	–	[122,123]
	Habituated	–	50	40	–	–	–	[132]
**Rwanda**	Total	182	–	400	–	–	–	[16,123,127]
	Habituated	182	–	Unknown	–	–	–	[16]
**Senegal**	Total	–	–	2,642	–	–	–	[125]
	Habituated	–	–	35	–	–	–	[132]
**Tanzania**	Total	–	–	2,500	–	–	–	[123]
	Habituated	–	–	150	–	–	–	[133]
**Uganda**	Total	414	–	4,950	–	–	–	[16,134,135]
	Habituated	75[1]	–	300	–	–	–	[136]

[1]Primates of Western Uganda.
[2]Amounts estimated by authors based on available published data.
